# Do delays between diagnosis and surgery in resectable oesophageal cancer affect survival? a study based on West Midlands cancer registration data

**DOI:** 10.1038/sj.bjc.6603333

**Published:** 2006-09-12

**Authors:** B S Kötz, S Croft, D R Ferry

**Affiliations:** 1 Division of Cancer Studies, University of Birmingham, Birmingham B15 2TT, UK; 2 West Midlands Cancer Intelligence Unit, University of Birmingham, Birmingham, B15 2TT, UK; 3 The Royal Wolverhampton Hospital NHS Trust, New Cross Hospital, Wolverhampton, UK

**Keywords:** oesophageal cancer, surgical resection, timing, delay, prognosis

## Abstract

This retrospective study investigates if delays between the diagnosis of cancer of the oesophagus and surgical resection influence long-term survival. Data held by the West Midlands Cancer Intelligence Unit on 800 patients who underwent oesophagectomy for a diagnosis of cancer of the oesophagus or oesophagogastric junction between 1995 and 2000 were reviewed. Six hundred and thirty-two patients treated with curative intention and who had not received neo-adjuvant treatment in the form of radio- or chemotherapy were included in the analysis. The time interval between histological diagnosis and surgical resection was stratified into four groups: less than 3, 3–6, 6–9 and more than 9 weeks. The Cox proportional hazard model was used to test for the independent effect of delays. The results showed no difference in long-term survival according to the delay between histological diagnosis and surgical resection. On multivariate analysis adverse prognostic factors were advanced age, incomplete resection and lymph node involvement. Patients with a longer delay had a higher rate of complete tumour resection suggesting that they were more appropriately selected for the surgical treatment approach. In conclusion we have found no evidence that shorter delays from the date of histological diagnosis to surgical resection are beneficial to long-term survival.

Almost 7400 new cases of oesophageal cancer were diagnosed in UK in 2000. In the same year about 6900 deaths owing to this type of cancer were registered. The outlook for most patients diagnosed with oesophageal cancer is poor with an overall 5-year survival of about 8%. Only about one-third of patients undergo surgical treatment as in the majority of patients the cancer is locally too advanced or has already metastasised at the time of diagnosis (McCulloch *et al*, 2003). Oesophagectomies carry one of the highest postoperative mortality among elective surgical procedures with a 30-day mortality of around 7% (O'Connell *et al*, 2004). Unfortunately surgery in itself is not very effective, with 50% of stage I patients and 80% of stage II patients dying within 5 years and an overall cure rate from the small fraction of operable patients being only around 20% (Gilbert *et al*, 2002; Gillison *et al*, 2002).

Not surprisingly several studies have shown that the ability to achieve a complete resection of the tumour with uninvolved margins on histological examination, defined by the American Joint Committee on Cancer Classification as ‘R0-Resection’, is a strong prognostic factor (Greene *et al*, 2002). Virtually no patients with either microscopically (R1) or macroscopically (R2) residual tumour are alive 5 years later, indeed most die within 2 years (Dexter *et al*, 2001; Hofstetter *et al*, 2002). Preoperative chemotherapy has been shown to reduce the number of involved lymph nodes, decrease the size of the tumour and improve the rate of complete resection in the responding group of patients (Kelson *et al*, 1998; Medical Research Council Oesophageal Working Party, 2002), which led to the evaluation of multimodality treatment approaches and the acceptance of neoadjuvant chemotherapy as the present standard of care.

Although some retrospective studies imply that delays between displaying first symptoms of the disease and establishing a diagnosis of oesophageal cancer may adversely affect outcome, no study has so far demonstrated a relation between short duration of symptoms and early tumour stage or an increased ability to achieve curative resection (Martin *et al*, 1997; Rothwell *et al*, 1997). Driven by public expectations and NHS waiting time targets there is increasing pressure to shorten the time from cancer diagnosis to treatment, but literature on the impact of any service or physician-related delay on outcome is scarce. The question if more timely surgery amongst those with a diagnosis could make a difference has not been addressed. In addition there are still concerns that a delay to surgical resection owing to treatment with neoadjuvant chemotherapy may be detrimental for the non-responding group of patients (Richel and Vervenne, 2004).

This retrospective study investigates if delays from the time of diagnosis to surgery impact on the long-term survival of patients undergoing oesophageal resection for squamous cell carcinoma (SCC) or adenocarcinoma (AC) of the oesophagus. All patients evaluated in this study did not receive neoadjuvant chemotherapy. As it would be ethically unacceptable to perform a prospective trial of early versus delayed surgery and because neoadjuvant chemotherapy is now regarded as the required standard of care for the majority of patients with resectable carcinoma of the oesophagus, future research is unlikely going to be able to address this question.

## METHODS

### Study design

Approval for the study was obtained from the Birmingham Local Research Ethics Committee. The West Midlands Cancer Intelligence Unit (WMCIU) was approached for identification of patients who underwent oesophagectomy for cancer of the oesophagus or oesophagogastric junction (OGJ) between 1995 and 2000 without having received neoadjuvant treatment in the form of chemotherapy or radiotherapy. These dates were chosen for two main reasons: first to allow for 5-year follow-up data and secondly because of a change of practice occurred in the UK towards giving neo-adjuvant chemotherapy following presentation of the results of the MRC OE02 Trial at the annual meeting of the American Society of Clinical Oncologists in May 2001 (Clark, 2001).

In accordance with WMCIU guidelines all treating surgeons were approached for permission to allow access to the records held by the WMCIU. The abbreviated patient files were reviewed and the following demographic and histopathological data collected: date of diagnosis (defined as the day on which the tumour biopsy was taken which resulted in the diagnosis of oesophageal cancer), age at diagnosis, treating hospital, date of surgery, tumour site, histological type and differentiation, pathological stage (pTNM), completeness of resection, patient's follow up status (alive or dead) and date of death if applicable.

The time delay to surgery was calculated from the date of diagnosis to the surgical resection date and categorised into four groups: 0–21 days, 22–42 days, 43–63 days and more than 64 days. Location of the tumour was defined as upper-, middle-, lower- third of the oesophagus or OGJ if the tumour was located between 14–18 cm, 19–34 cm, 35–40 cm and more than 40 cm from the incisors, respectively. Tumours were coded as ‘not otherwise specified’ if the exact location could not be elicited. Tumours located at the cardia or corpus of the stomach without involvement of the lower oesophagus on histology, were disregarded as stomach cancer. The tumour differentiation was taken from the histopathology report and was recorded according to the predominant area as well-, moderately- or poorly-differentiated (Ibrahim, 2000). If the tumour was described as ‘well to moderately’ or ‘moderate to poorly’ differentiated, the poorest tumour differentiation was taken. The tumour, node and metastasis classification according to the pathological report (pTNM) was recorded (Sobin and Wittekind, 2002). Completeness of resection was evaluated from the histopathology report and any surgical notes and classified as either ‘complete resection’ (R0) or ‘microscopically or macroscopically incomplete resection’ (R1/R2). Date of death was recorded as per death certificate. Median and overall survival was calculated from the date of diagnosis. Patients alive on the 1 September 2004 were censored on that date.

### Data analysis

Baseline differences in prognostic or possible confounding factors between the four different treatment delay groups were performed by the *χ*^2^-test. Survival curves were calculated using the Kaplan–Meier method and differences assessed by log-rank statistics. The relationship between survival and the following prognostic factors were evaluated by univariate analysis: age (less than 60 years, 60–70 or more than 70 years), gender, hospital type (University Hospitals or District General Hospitals (DGH)), histological type (AC or SCC), lymph node involvement (lymph node positive or negative), location of tumour, differentiation, completeness of resection and time interval from diagnosis to treatment (all as defined above). The Cox proportional hazard model was used to test for the independent effect of delays after adjusting for the above variables and hazard ratios for survival were estimated with 95% confidence limits. Statistical analysis was performed with SSPS for Windows Version 11.5.

## RESULTS

### Patients

The WMCIU identified 1260 patients with a diagnosis of oesophageal cancer or cancer of the cardia who had a surgical intervention for this diagnosis between 1995 and 2000. The patients were registered under eight cardio-thoracic- and 21 general surgeons, two gastro-enterologists and general general physicians. All but two general surgeons who had retired were approached for permission to utilise cancer registry information for the purpose of this study. Twenty consultants (64.5%) responded and confirmed their agreement.

The records of 800 patients were reviewed. Patients were excluded from further analysis for the following reasons: Surgery not with curative intention: 52 patients; surgery for local recurrent disease: one patient; treatment with neo-adjuvant chemotherapy in MRC OE 02 Study (patients allocated to the surgery only arm were included in the analysis): 17 patients; cancer located in the cardia without involvement of the OGJ on histopathological examination: 22 patients; histology other than SCC or AC: 14 patients; date of the upper gastro-intestinal endoscopy or original biopsy missing: 61 patients. All remaining 632 patients were included in the analysis. Their demographic and tumour characteristics are described in Table 1.

The male-to-female ratio was 2.5–1. Their median age was 67 years (range 21–89 years). About half of the patients were operated in University Hospitals. In half of the patients the tumour was located at the OGJ and in another third at the lower oesophagus. The predominant histological type was AC and the majority of tumours were moderate or poorly differentiated. The commonest pathological tumour stage was pT_3_N_1_ (Stage III) in 338 patients (53%), the second commonest was pT_2 or 3_ N_0_ (Stage IIA) in 159 patients (25%). In one-third of all oesophagectomies no lymph nodes were involved and a complete resection was achieved in 68% of operations.

### Delays

**Figure 1:**
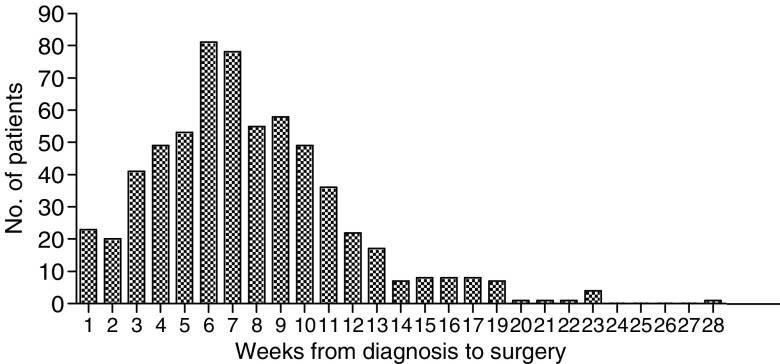
Bar chart illustrating time interval from histological diagnosis to surgical resection.

**Figure 2:**
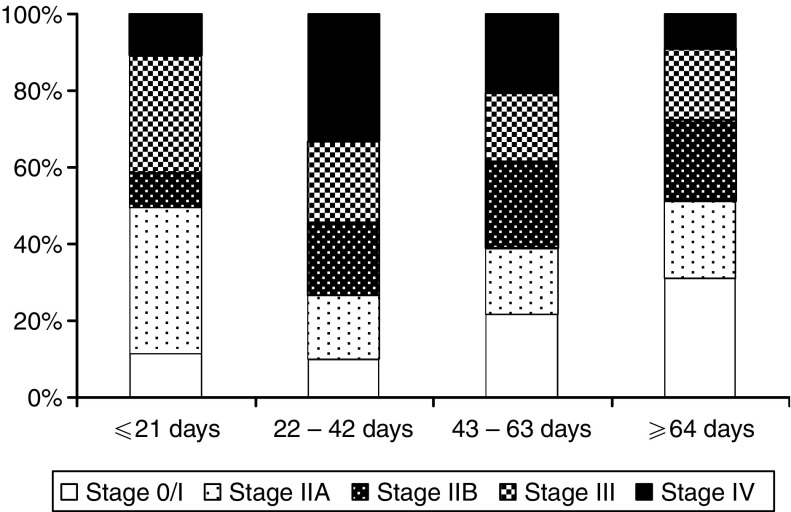
Percentage bar chart showing the distribution of disease stage according to the time interval from histological diagnosis to surgical resection.

The median time from diagnostic endoscopy to surgical resection was 47 days and ranged from 0 to 287 days (Figure 1). Thirteen percent of patients were operated within 3 weeks, 39% within 6 weeks, 72% within 9 weeks and 90% within 12 weeks. Patients operated later after their diagnosis were of more advanced age (*P*=0.0255) and more likely operated at a University Hospital (*P*<0.0001) than those treated sooner. On histology tumours resected after a longer delay were more likely to be ACs (*P*=0.0008) and more likely to have been completely resected (*P*=0.0097) compared to tumours resected after a shorter delay (Table 2). Patients operated after a longer delay were less likely to have pathological stage IV disease (*P*=0.012) (Figure 2). There was no difference between complete resection rates at operations performed at University hospitals and DGHs (70% and 66%, respectively, *P*=0.269). Patient's gender, postoperative mortality, tumour location, tumour grade or lymph node status were not significantly different in the four different delay groups.

### Survival

Five hundred and thirty-nine patients (85%) were registered dead and 93 patients censored on the 1 September 2004 for the Kaplan–Meier survival analysis. Median follow-up was 6.5 years, range 3.7–9.7 years. The median survival of all patients was 15.4 months and the 2-, 3 and 5-year survival rates were 33, 24 and 17.5%, respectively.

**Figure 3:**
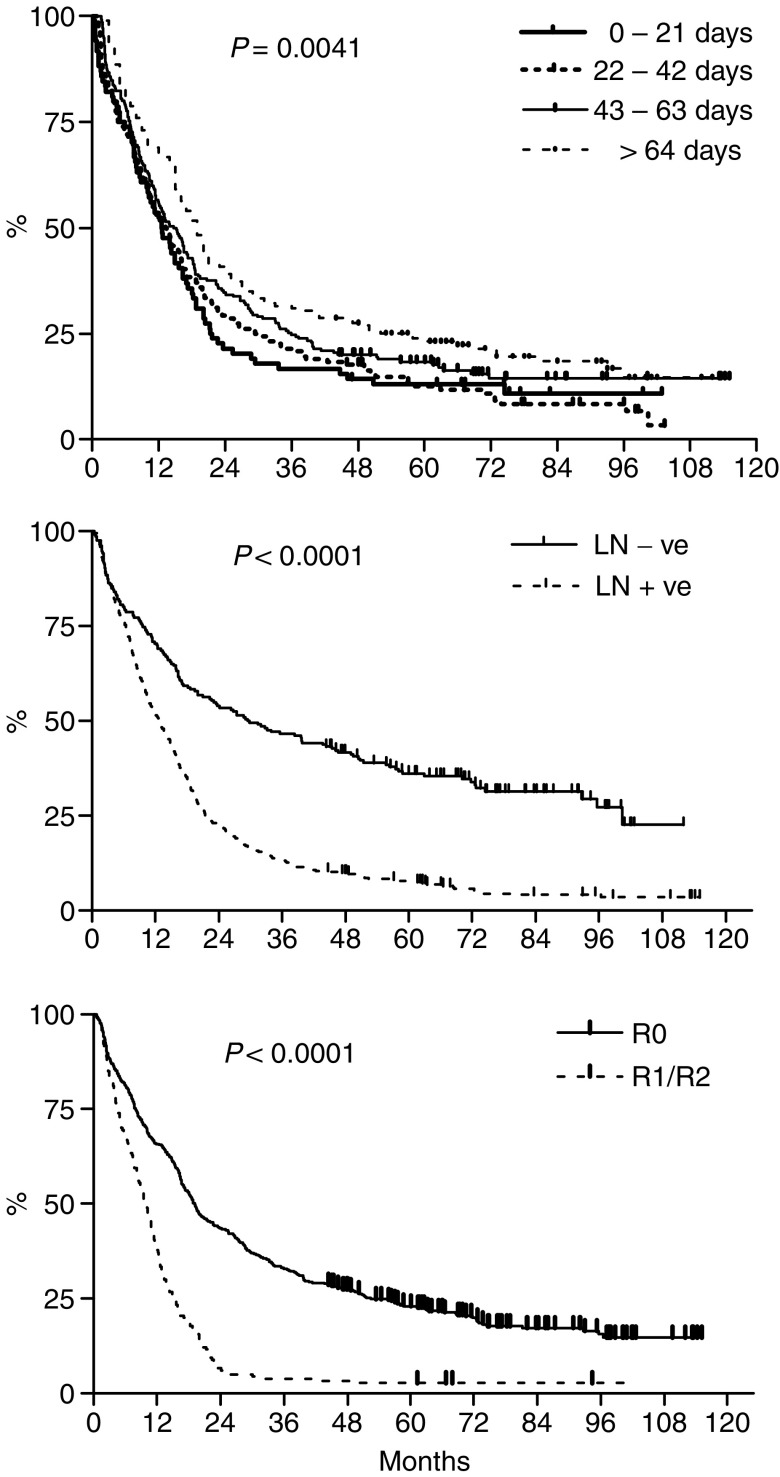
Kaplan–Meier survival curves of 632 patients with cancer of the oesophagus or OGJ according to interval from histological diagnosis to surgical resection (top), lymph-node status (middle) and completeness of resection (‘R0’ – complete resection without involved margins on histology, ‘R1/R2’ – micro- or macroscopically incomplete resection) (bottom).

On univariate analysis a younger age at diagnosis, a well-differentiated tumour, absence of lymph node involvement, complete resection and a longer delay between diagnosis and surgical resection were all associated with improved survival (Table 3 and Figure 3). There were no significant differences in median survival according to patients' gender, the hospital type at which the surgery was performed, tumour location or histological tumour type. Multivariate analysis revealed that a longer delay between diagnosis and surgery was not an independent prognostic factor, whereas a significant survival benefit persisted for patients of younger age, complete resection of the tumour and absence of lymph-node involvement (Table 4).

## DISCUSSION

The time interval between diagnosis and surgery was with a median delay of 47 days (6. 7 weeks) comparatively longer than reported in previous studies. Martin *et al* (1997) examined delays in patients with oesophagogastric cancer presenting to the surgical department of a large teaching hospital in the UK and found a median delay of 3.9 weeks and the prospective Scottish audit of gastric and oesophageal cancer reported a delay of more than 1 month from establishing a histological diagnosis to surgical resection in 45% of patients, whereas in this study 76% of patients waited longer than 4 weeks.

Despite the relatively large difference in time interval between diagnosis and surgical resection in this retrospective study ranging from less than 3 weeks to more than 9 weeks, we did not find any detrimental effect of delays on survival. The most significant adverse prognostic factors influencing long-term survival in the patient group examined were involvement of local lymph nodes and the inability to achieve a complete resection, confirming results of previous studies (Lieberman *et al*, 1995; Dexter *et al*, 2001; Butsch *et al*, 2002; Hofstetter *et al*, 2002).

What is commonly described and perceived by health authorities as ‘waiting time’ between diagnosis and treatment, constitutes a combination of important steps in the decision-making processes leading to recommendation of the best therapeutic approach for an individual patient. This process generally includes thorough evaluation of the extent of the disease through staging investigations, assessment of the patients’ co-morbidity and multidisciplinary case discussion. A relation between preoperative staging strategy employed and survival has been observed in previous studies. Hofstetter *et al* (2002) noted in their report on treatment outcomes of resected oesophageal cancer that the group of patients who were preoperatively staged with endoscopic ultrasound had a significantly improved survival. Gilbert *et al* (2002) found in the Scottish audit of gastric and oesophageal cancer 1997–2000 that patients in whom no regional disease was found with a combination of CT and laparoscopy or endoscopic ultrasound had significantly improved survival post surgery.

Although reasons for a delay between diagnosis and surgery or the type of staging investigations used could not be explored in this study, treatment delays are likely multifactorial rather than simply delays in service provision. In this study, younger patients were operated sooner after their diagnosis, but were also more likely to have had only an incomplete resection of the tumour. Patients with a longer time interval between tumour biopsy and surgical resection were less likely to have disease that had metastasised beyond regional lymph nodes, when surgery can only be regarded as palliative. It is important to note that with a median survival of only 9.8 months patients whose tumour could not be completely excised had a similar poor prognosis than patients who receive only palliative treatment (Frenken, 2001; Ross *et al*, 2002).

We conclude that patients who waited longer for their operation were more appropriately selected for the surgical treatment approach. Recording of the staging investigations used before surgery would give valuable information for future research. Regarding the use of neo-adjuvant chemotherapy, we found that delays equivalent to the time required to give two cycles of chemotherapy will not adversely affect the chance of cure and emphasise that neo-adjuvant chemotherapy is now the accepted standard of care for most patients with this type of cancer.

## Figures and Tables

**Table 1:** Characteristics of all patients (percentages in brackets)

**Characteristics**	**Number of patients (*n*=632)**
*Age (years)*	
<60	171 (27.1)
60–70	229 (36.2)
>70	232 (36.7)
	
*Gender*	
Male	451 (71.4)
Female	181 (28.6)
	
*Hospital type*	
University hospitals	326 (51.6)
DGHs	289 (45.7)
PrH	17 (2.7)
	
*Tumour location*	
Upper 1/3 of oesophagus	4 (0.6)
Middle 1/3 of oesophagus	71 (11.2)
Lower 1/3 of oesophagus	210 (33.2)
Oesophago-gastric junction	324 (51.3)
Not specified	23 (3.6)
	
*Histology*	
AC	475 (75.2)
SCC	157 (24.8)
	
*Tumour differentiation*	
Well	57 (9)
Moderate	237 (37.5)
Poor	300 (47.5)
Not specified	38 (6)
	
*Stage*	
0	5 (0.8)
I	43 (6.8)
II A	159 (25.2)
II B	43 (6.8)
III	338 (53.5)
IV	39 (6.2)
Not specified	5 (0.8)
	
*LN status*	
LN –ve	210 (33.2)
LN +ve	417 (66)
Not specified	5 (0.8)
	
*Resection*	
R0	432 (68.4)
R1/2	180 (28.4)
Not specified	20 (3.2)
	
*Postoperative mortality*	
30-Day	75 (11.8)
90-Day	117 (18.5)

Abbreviations: AC, adenocarcinoma; DGH, district general hospital; LN, lymph node; OGJ, oesophagogastric junction; Prh, private hospital; SCC, squamous cell carcinoma.

**Table 2:** Distribution of prognostic factors according to delay form diagnosis to surgery (percentages in brackets)

**Characteristic**	***P*-value**	**<21 days (n=84)**	**22–42 days (n=164)**	**43–63 days(n=210)**	**>64 days (n=174)**
*Age (years)*	**0.0255**				
<60		31 (36.9)	54 (32.9)	49 (23.3)	37 (21.3)
60–70		24 (28.6)	62 (37.8)	80 (38.1)	63 (36.2)
>70		29 (34.5)	48 (29.3)	81 (38.6)	74 (42.5)
					
*Gender*	0.3681				
Male		54 (64.3)	123 (75)	149 (71)	125 (71.8)
Female		30 (35.7)	41 (25)	61 (29)	49 (28.2)
					
*Hospital type*	**<0.0001**				
UHs		31 (36.9)	74 (45.1)	107 (51)	115 (66)
DGHs		45 (53.6)	84 (51.2)	101 (48.1)	58 (33.4)
PrHs		8 (9.5)	6 (3.7)	2 (0.9)	1 (0.6)
					
*Tumour location*	0.054				
Upper/middle		15 (17.9)	19 (11.6)	27 (12.9)	14 (8.1)
Lower		35 (41.7)	54 (32.9)	69 (32.9)	52 (29.9)
OGJ		32 (38)	81 (49.4)	107 (50.9)	104 (59.8)
Not specified		2 (2.4)	10 (6.1)	7 (3.3)	4 (2.2)
					
*Histology*	**0.0008**				
AC		54 (64.3)	118 (72)	154 (73.3)	149 (85.6)
SCC		30 (35.7)	46 (28)	56 (26.7)	25 (14.4)
					
*Tumour grade*	0.1699				
WD		7 (8.4)	13 (7.9)	15 (7.1)	23 (13.2)
MD		37 (44)	64 (39)	81 (38.6)	55 (31.6)
PD		39 (46.4)	77 (47)	103 (49.1)	81 (46.6)
Not specified		1 (1.2)	10 (6.1)	11 (5.2)	15 (8.6)
					
*Lymph-node status*	0.0556				
LN −ve		32 (38.1)	41 (25)	70 (33.3)	68 (39.1)
LN +ve		52 (61.9)	123 (75)	137 (65.3)	104 (59.7)
Not specified		0	0	3 (1.4)	2 (1.1)
					
*Resection*	**0.0097**				
R0		55 (65.5)	99 (60.4)	153 (72.9)	125 (71.8)
R1/2		28 (33.3)	63 (38.4)	49 (23.3)	41 (23.6)
Not specified		1 (1.2)	2 (1.2)	8 (3.8)	8 (4.6)
					
*Postoperative mortality*	0.9716				
30-day		11 (13)	19 (11.6)	24 (11.4)	22 (12.6)
90-day		16 (19)	33 (20.1)	37 (17.6)	32 (18.4)

Abbreviations: AC, adenocarcinoma; DGH, district general hospital; LN, lymph node; OGJ, oesophagogastric junction; Prh, private hospital; SCC, squamous cell carcinoma; UH, university hospitals.

Significant *P*-values in bold.

**Table 3:** HRs with 95% confidence intervals from univariate analysis: median survival according to individual prognostic factors and delay to surgical resection

**Characteristics**	**Median survival (months)**	***P*-value**	**HR**	**95% CI**
*Delay (days)*		**0.0041**		
0–21	12.6		1	
22–42	13.29		0.962	(0.726–1.274)
43–63	15.19		0.817	(0.607–1.074)
>64	19		0.654	(0.454–0.845)
				
*Age (years)*		**0.04**		
<60	18.02		1	
60–70	14.86		1.26	(1.017–1.558)
>70	13.61		1.305	(1.053–1.614)
				
*Gender*		0.354		
Female	16.04		1	
Male	15.29		1.093	(0.907–1.314)
				
*Hospital type*		0.926		
UHs	16.04		1	
DGHs	14.76		1.008	(0.849–1.197)
				
*Tumour location*		0.369		
Upper+middle	12.56		1	
Lower	15.19		0.862	(0.641–1.145)
OGJ	16.24		0.825	(0.614–1.080)
				
*Histology*		0.578		
AC	15.52		1	
SCC	14.93		1.057	(0.868–1.289)
				
*Tumour grade*		**0.025**		
WD	23.54		1	
MD	16.41		1.185	(0.871–1.588)
PD	12.76		1.437	(1.054–1.828)
				
*LN status*		**<0.0001**		
LN –ve	29.6		1	
LN +ve	12.76		2.268	(1.847–2.624)
				
*Resection*		**<0.0001**		
R0	19.1		1	
R1/2	9.86		2.324	(2.474–3.921)

Abbreviations: AC, adenocarcinoma; CI, confidence interval; DGH, district general hospital; HR, hazard ratio; LN, lymph node; OGJ, oesophagogastric junction; Prh, private hospital; SCC, squamous cell carcinoma; UH, university hospitals.

Significant *P*-values in bold.

**Table 4:** HR ratios with 95% CIs from multivariate analysis

**Characteristics**	***P*-value**	**HR**	**95% CI**
*Delay (days)*	0.113		
0–21		1	
22–42		1.042	(0.774–1.402)
43–63		0.864	(0.638–1.170)
>64		0.782	(0.574–1.066)
			
*Age (years)*	**<0.0001**		
<60		1	
60–70		1.393	(1.103–1.760)
>70		1.746	(1.380–2.209)
			
*Gender*	0.129		
Female		1	
Male		1.186	(0.952–1.478)
			
*Hospital type*	0.833		
UHs		1	
DGHs		1.033	(0.584–1.825)
			
*Tumour location*	0.608		
Upper+middle		1	
Lower		0.895	(0.655–1.222)
OGJ		0.837	(0.588–1.191)
			
*Histology*	0.148		
AC		1	
SCC		1.224	(0.931–1.611)
			
*Tumour grade*	0.388		
WD		1	
MD		1.06	(0.762–1.474)
PD		1.201	(0.865–1.669)
			
*LN status*	**<0.0001**		
LN −ve		1	
LN +ve		1.982	(1.598–2.458)
			
*Resection*	**<0.0001**		
R0		1	
R1/2		2.118	(1.712–2.621)

Abbreviations: AC, adenocarcinoma; CI, confidence interval; DGH, district general hospital; HR, hazard ratio; LN, lymph node; OGJ, oesophagogastric junction; Prh, private hospital; SCC, squamous cell carcinoma; UH, university hospitals.

Significant *P*-values in bold.
